# MISSEL: a method to identify a large number of small species-specific genomic subsequences and its application to viruses classification

**DOI:** 10.1186/s13040-016-0116-2

**Published:** 2016-12-06

**Authors:** Giulia Fiscon, Emanuel Weitschek, Eleonora Cella, Alessandra Lo Presti, Marta Giovanetti, Muhammed Babakir-Mina, Marco Ciotti, Massimo Ciccozzi, Alessandra Pierangeli, Paola Bertolazzi, Giovanni Felici

**Affiliations:** 1 Institute of Systems Analysis and Computer Science A. Ruberti (IASI), National Research Council (CNR), Via dei Taurini 19, Rome, 00185 Italy; 2 Department of Engineering, Uninettuno International University, Corso Vittorio Emanuele II 39, Rome, 00186 Italy; 3 Department of Infectious Diseases, Istituto Superiore di Sanita, Viale Regina Margherita 299, Rome, 00161 Italy; 4 Public Health and Infectious Diseases, Sapienza University, Piazzale Aldo Moro 5, Rome, 00185 Italy; 5 Department of Biology, University of Rome Tor Vergata, Via della Ricerca Scientifica 1, Rome, 00133 Italy; 6 Foundation of Technical Education, Sulaimaniyah, Kurdistan Region, Iraq; 7 Laboratory of Molecular Virology, Polyclinic Tor Vergata Foundation, Viale Oxford 81, Rome, 00133 Italy; 8 Virology Laboratory, Department of Molecular Medicine, Sapienza University, Viale di Porta Tiburtina 2, Rome, 00185 Italy

**Keywords:** Classification of genomic sequences, Genetic algorithms, Supervised learning, Extraction of multiple classification models

## Abstract

**Background:**

Continuous improvements in next generation sequencing technologies led to ever-increasing collections of genomic sequences, which have not been easily characterized by biologists, and whose analysis requires huge computational effort. The classification of species emerged as one of the main applications of DNA analysis and has been addressed with several approaches, e.g., multiple alignments-, phylogenetic trees-, statistical- and character-based methods.

**Results:**

We propose a supervised method based on a genetic algorithm to identify small genomic subsequences that discriminate among different species. The method identifies multiple subsequences of bounded length with the same information power in a given genomic region. The algorithm has been successfully evaluated through its integration into a rule-based classification framework and applied to three different biological data sets: Influenza, Polyoma, and Rhino virus sequences.

**Conclusions:**

We discover a large number of small subsequences that can be used to identify each virus type with high accuracy and low computational time, and moreover help to characterize different genomic regions. Bounding their length to 20, our method found 1164 characterizing subsequences for all the Influenza virus subtypes, 194 for all the Polyoma viruses, and 11 for Rhino viruses. The abundance of small separating subsequences extracted for each genomic region may be an important support for quick and robust virus identification.

Finally, useful biological information can be derived by the relative location and abundance of such subsequences along the different regions.

**Electronic supplementary material:**

The online version of this article (doi:10.1186/s13040-016-0116-2) contains supplementary material, which is available to authorized users.

## Background

The analysis of DNA sequences of living organisms is relevant for many genetic, biological, and medical purposes. It can support the automated recognition and classification of organisms, genomic regions characterization, and the study of genetic evolution of population of individuals of the same species. Its deployment for taxonomic classification of organisms is first proposed for archaea, bacteria, protists and viruses [1–3], and then for many other domains [4, 5] becoming a central research topic in Bioinformatics.

DNA sequences used for this aim are different for diverse organisms and corresponds to specific *genomic regions*, e.g., the mitocondrial DNA region associated with cytochrome c oxidase I (COI), commonly refered to as *Barcode*, largely used for species classification in the animal kingdom (see, among others, [6]).

The methods that are adopted to pursue this task are inherited from the more general sequence analysis and supervised learning (i.e., classification) literature, and can be divided into 3 main groups [7]: tree-based methods, similarity-based methods, and character-based methods. The first ones make use of Parsimony (i.e., PAR [8]), or Neighbor Joining (i.e., NJ [9]), or Bayesian Inference [10] to assign unidentified sequence query to categories based on their clusters membership in a tree. The second group is composed of methods such as BLAST [11], NN [12], TaxonDNA [13] that assign sequence queries to categories based on how many nucleotide characters they share. The third group of methods comprises tools like DNA-BAR [14], BLOG [15], CAOS [16], BRONX [17], PTIGS-IdIt [18], Linker [19], and Alignment-free analytics [20, 21]. Methods in the latter group are based on the identification of specific and limited nucleotides positions of the DNA sequences that can be used for recognizing the class of the sample sequences.

All the mentioned approaches share the same supervised learning paradigm: a set of sample sequences for which the class is known (the so-called *training* set, composed of *labeled* sequences) is analyzed to derive rules that may recognize also unlabeled sequences [22, 23].

Supervised learning is typically effective when the domain of the samples is well defined, e.g., we know that the samples can belong to a known number of species or classes; good examples can be found in [24], where supervised learning methods successfully classify species through the analysis of DNA *Barcode* sequences.

The literature offers indeed several methods to identify virus species through the analysis of DNA sequences (among them, [25–29]).

In this paper, we investigate some aspects that remained unexplored: in particular, where the nucleotide changes that make one species different from another are located, if such nucleotides are close to each other along the sequence, how many equivalent characterizing subsequences are within the boundaries of the considered sequence. An additional aspect, that will be considered also in this paper, is the differential analysis of known genomic regions, to see if they show a different abundance of genetic variations with respect to a number of taxa.

Based on these considerations, we address a different and original problem: within large and well-defined DNA sequences, identify small subsequences that contain all the information needed to classify new sequences with a good level of precision, and to measure the abundance of such subsequences, their location, and their concentration in the original sequences. Solving such a problem contributes to the characterization of the different portions of the sequences, generating new insight on the role of certain genomic regions in species characterization. As shown in the last section of this paper, this spatial information can be very relevant, and can be associated with biological properties of the original sequence.

We design MISSEL (Multiple SubSequences Extractor for cLassification) - a new method to solve this problem based on a meta-heuristic algorithm, which identifies many small subsequences that retain enough information to classify the considered species. Such subsequences are used to identify portions of the sequences that are rich of discriminating information with respect to those that are non-meaningful.

We apply MISSEL to the genomes of Rhino, Influenza, and Polyoma viruses. These three virus types, having positive and negative strand RNA or DNA genomes respectively, present features that make them particularly suited for the proposed method.

The recent discovery of novel and divergent human Polyoma viruses raises key questions regarding their evolution, tropism, latency, reactivation, immune evasion and contribution to diseases. Moreover, the increasing number of human and animal Polyoma viruses suggests that many of them remain to be unveiled. The most common pathological manifestation is the reactivation in an immunosuppressed host, so that a molecular characterization is often needed.

Influenza subtyping is of epidemiological and clinical significance, beside the obvious interest during pandemic periods. In particular, seasonal Influenza A(H3N2) generally causes more severe outcomes among at risk groups than A(H1N1) or Influenza B viruses [30]. For that reason, Influenza epidemic surveillance in Europe has been recently implemented with virological surveillance to alert about the real-time predominating subtype.

As far as human Rhino virus (HRV) is concerned, conflicting reports have associated (or not) HRV-C to a clinical severity greater than HRV-A and to recurrent wheezing [31, 32], so that genotyping should be implemented to possibly clarify these issues.

In all three cases, it is important to classify with precision the different virus types, but also to identify the regions that mostly support such classification, which are supposedly the regions where mutations have occurred in the evolution process. The number and the size of separating subsequences provide additional insight on the location of mutations and on their uniques in characterizing the analyzed taxa.

In these data sets MISSEL identified 1164 equivalent small subsequences for all the Influenza virus subtypes, 194 for the Polyoma viruses, and 11 for the Rhino viruses. The size of such subsequences is rather small, as their are composed by no more than 20 contiguous nucleotides.

Below we provide a clear statement of the problem and a description of the proposed method for its solution. A compact additional analysis of the related literature in the field of meta-heuristic algorithms is also provided.

### Problem statement

Given a set of genomic sequences belonging to different classes (i.e., species), find the largest possible number of subsequences with the following properties:

- The values of the nucleotides (i.e., A,C,G,T/U) in the positions belonging to the subsequence can be used to derive rules that can predict the class of new sequences with high precision (i.e., considering the genomic region LT “if *pos*_435_=*C* AND *pos*_436_=*T*, then the virus is a Polyoma HP9”);
- The size of the subsequence is bounded and small if compared to the size of the input sequences;
- The subsequence does not contain any other subsequence with the same properties.

### Related work

The above problem contains several complex aspects. The main one is that searching for many subsequences with desirable properties is much more difficult than searching for a single optimal one. Additionally, the dimensions of the problem to be solved are typically very large (i.e., DNA sequences with thousands of bases). The complexity of the problem does not suggest a straightforward deployment of a mathematical optimization model, and therefore we consider a meta-heuristic approach that is much faster than enumeration, and sufficiently precise and time-effective.

*Meta-heuristics* are nature-inspired algorithms that can be suitably customized to solve complex and computationally hard problems, and can be inspired to different principles, such as *Ant colony optimization* [33], *Genetic Algorithms* [34], *Simulated annealing* [35], *Tabu Search* [36], *Particle swarm optimization* [37]. Several authors in the literature considered similar problems, although they cannot be reconducted to the framework of multiple solutions that we adopt here.

Recent studies [38–40] focused on problems with multiple objective functions, often used as a tool to counterbalance the measurement bias affecting solutions based on a single objective functions, or to mitigate the effect of noise in the data. Deb et al. [41] also approached the issue of identifying gene subsets to achieve reliable classification on available disease samples by modeling it as a multi-objective optimization problem. Furthermore, they proposed a multimodal multi-objective evolutionary algorithm that finds multiple, multimodal, non-dominated solutions [42] in one single run. Those are defined as solutions that have identical objective values, but differ in their phenotypes. Other works [43, 44] pointed to multiple membership classification, dealing with the fitting of complex statistical models to large data sets. Again, Liu et al. [45] proposed a subset gene identification consisting of multiple objectives, but, differently from Deb et al. [41], they scalarize the objective vector into one objective that is solved by using a parallel genetic algorithm, in order to avoid expensive computing cost. Kohavi et al. [46] addressed the problem of searching for optimal gene subsets of the same size, emphasizing the use of wrapper methods for the features selection step. Rather than trying to maximize accuracy, they identified which features were relevant, and used only those features during learning. The goal of our work is again different: to extract information on interesting portions of the genomic sequences by taking into account equivalent subsequences.

The rest of the paper is organized as follows: in Section “Materials and methods”, we provide a detailed description of the algorithm. In Section “Results and discussion”, we report and discuss the application of our algorithm to extract equivalent and multiple subsequences from three experimental data sets of virus sequences, described at the beginning of that section, and we describe the results of the classification analysis of the species of those samples. Finally, in Section “Conclusions”, we delineate the conclusions of the work both from the algorithmic and biological point of view jointly with its future extensions.

## Materials and methods

The main components of our work are described below, starting from a detailed description of the algorithm.

### MISSEL: multiple subsequences extractor for classification

In this section, we present MISSEL (Multiple SubSequences Extractor for cLassification), a method to extract alternative and equivalent subsequences that can be applied in supervised classification problems for biological sequences belonging to different classes. A subsequence is a set of consecutive nucleotide positions of the sequence. Given a set of aligned sequences of equal length *n*, belonging to different classes, we look for the largest number of subsequences with the following characteristics:

- They are *separating subsequences*, i.e., knowing the nucleotide in the positions of the subsequence allows one to predict, with high reliability, the class of sequences whose class is unknown;
- The length of the subsequence (also referred to as its *size*) is small and anyway not larger than a given threshold.

MISSEL is an ad-hoc nature-inspired meta-heuristics based on an evolutionary approach [34], which identifies the desired subsequences in a reasonable computational time. In the following, we discuss the details of the algorithm.

### Genetic algorithm

MISSEL is based on an ad-hoc developed Genetic Algorithm (GA). It implements a search paradigm that exploits an ever-changing population whose individuals represent possible solutions of the given problem. Such a population evolves according to a set of *genetic operators*.

Each individual *s*_*i*_ (i.e., candidate solution) is a set of consecutive positions of the input sequences. To each individual, the following values are associated:

- *β*_*i*_, its length;
- *σ*_*i*_, its starting position;
- *α*_*i*_, its discriminant power (described in detail in the following);
- $n_{\alpha _{i}}$, number of pairs of sequences of different class not covered at level *α*_*i*_ (better explained in the following);
- *F*(*s*_*i*_), its fitness value (better explained in the following).

Firstly, a *population* *S*_0_ of candidate individuals is generated and initialized (at time *t*=0). Then, the population evolves (*t*>0), selecting each individual according to a randomized rule based on its fitness value, and computing a new individual by means of different *genetic operations*. At each iteration, the survival capacity of each *i*-th individual of the population is defined according to its *fitness* value, so that the new population will have improved overall fitness. New generations of the population are iteratively computed until one stopping criterion is verified. Once the genetic algorithm has been run, the best individuals are returned.

#### Population and fitness

At the *t*-th iteration of our scheme a population *S*_*t*_ is available.

We recall that a subsequence is fully identified by *β*_*i*_ and *σ*_*i*_, and it is also referred to as an *individual* of the population. A generic individual in *S*_*t*_ is referred to as *s*_*i*_. We note that two individuals (*i* and *j*) with same starting position (*σ*_*i*_=*σ*_*j*_) and same length (*β*_*i*_=*β*_*j*_) identify exactly the same positions and are therefore equal.

Additionally, we refer to *R* as the repository of individuals that are candidate for being part of the final solution. The discriminant power of *s*_*i*_ (referred to as *α*_*i*_) is derived from a feature selection model based on an integer program already discussed in [47–50]: for a set of features of a given size, it indicates a lower bound on how many times a pair of samples of different classes is separated by a feature in the set. Intuitively, such a value represents how many different discriminating models can be build with the considered features. In this case, the features are the positions of the small subsequence that corresponds to the individual. Additionally, we also take into account the value $n_{\alpha _{i}}$, that indicates how many pairs of samples of different classes are separated exactly by *α*_*i*_ features. We observe that individuals with a high value of *α*_*i*_ are very rich of discriminating information; moreover, a small value of $n_{\alpha _{i}}$ indicates that the value of *α*_*i*_ may be further increased with few additional features (for this reason, *α*_*i*_ and $n_{\alpha _{i}}$ are the main ingredient of the fitness function of *s*_*i*_, described below). Given an individual *s*_*i*_ identified by its length *β*_*i*_ and its starting point *σ*_*i*_, the computation of *α*_*i*_ is straightforward: build a matrix with *m* rows indexed by the pairs of samples belonging to different classes and *n* columns indexed by the positions in *s*_*i*_; then, each element of this matrix shows the value 1, if the value of the nucleotide in the position indexed by the column is different from the pair indexed by the row, and 0 otherwise. The row-wise minimum number of ones for the *β*_*i*_ columns that start from *σ*_*i*_ is exactly *α*_*i*_. To complete this description, we note that *α*_*i*_≤*β*_*i*_ and that $n_{\alpha _{i}} \leq K$, where *K* is the number of pairs of input sequences that belong to different classes.

With the above ingredients, the fitness function *F*(*s*_*i*_) can be computed, based on the value of *α*_*i*_ (to which fitness is directly related), *β*_*i*_ and $n_{\alpha _{i}}$ (fitness being indirectly related with both of them). Then, *F*(*s*_*i*_) takes into account:

- The *quality* of individual *s*_*i*_ (related with $\alpha _{i} \over \beta _{i}$, the larger, the better).
- The *size* of individual *s*_*i*_ (*β*_*i*_, the smaller, the better);

using the following formula:

1 $$  F(s_{i}) = \omega_{A}\cdot A + \omega_{B}\cdot B  $$

where:

2 $$  A = \frac{\alpha_{i}}{\beta_{i}} + \frac{\left(K - n_{\alpha_{i}}\right)}{K}  $$

3 $$  B = \frac{n-\beta+1}{n}  $$

*A,B*∈ [0,1], *ω*_*A*_ and *ω*_*B*_ are weights for the terms *A* (2) and *B* (3) of *F*, respectively.

The complete list of parameters used in the algorithm is provided in Table 1. Given the parameters of Table 1, we can specify that *s*_*i*_∈*S*_*t*_, with *i*=1,⋯,*I*, |*I*|∈[Initipop,Dimstore] and *t*∈[0,Maxiter]. The algorithm terminates when one of the following stopping criteria is verified: (i) the fixed number of iterations exceeds the value of Maxiter; (ii) the number of individuals exceeds the value of Dimstore; (iii) the number of extracted individuals does not change anymore.

**Table 1:** Overview of the parameters of the genetic algorithm

Parameter	Description
Maxiter	maximum number of iterations
Max _*β*_	maximum length of the subsequences
Base _*σ*_	starting value of *σ*
Dimstore	maximum cardinality of *R*
Initpop	cardinality of the initial population

#### Relationship among individuals: equivalence and dominance

Among all the computed subsequences, we focus on the *equivalent* and *non-dominated* ones. Let *S* be the set of individuals in the repository *R*, and consider the following definitions:

##### **Definition 1**

**Equivalent individuals** Given two individuals *s*_1_,*s*_2_∈*S*,they are equivalent if the following 3 conditions are verified: 1) *α*_1_=*α*_2_; 2) *β*_1_=*β*_2_; 3) *σ*_1_≠*σ*_2_.

two equivalent individuals should not be both stored in *R*. We now turn to consider a *dominance* relation between individuals.

##### **Definition 2**

**Domination between individuals** *Given two individuals* *s*_1_,*s*_2_∈*S*, *we say that* *s*_1_ *dominates* *s*_2_ *(* *s*_1_≻*s*_2_ *) if one of the following 2 conditions is verified: 1)* *α*_1_>*α*_2_ *and* *β*_1_≤*β*_2_; *2)* *α*_1_≥*α*_2_ *and* *β*_1_<*β*_2_.

As a consequence, we have that individual *s*_*i*_∈*S* is *non-dominated* if there is no individual *s*_*j*_∈*S,s*_*j*_≠*s*_*i*_, such that *s*_*j*_≻*s*_*i*_.

Less formally, individual *i* dominates individual *j* if it has the same length (*β*_*i*_=*β*_*j*_), but it shows a higher separation power (*α*_*i*_>*α*_*j*_), or if it has the same value of separation power (*α*_*i*_=*α*_*j*_), but it has a shorter length (*β*_*i*_<*β*_*j*_). Our algorithm computes all the *equivalent* individuals by filtering out any individual that is dominated by another one in *R*. Moreover, if a new individual is equivalent to an individual already in *R* can be computed in constant time.

The quality of the final subset of solutions mostly depends on the way the genetic algorithm is implemented; that is, how the fitness of each solution is measured and how subsequences are selected and extended in each iteration. The main idea is that, when properly designed, the genetic algorithm can determine populations that are sufficiently heterogeneous and whose solutions have good values.

#### Genetic operations

We summarize below the genetic operators adopted in MISSEL.

**Selection**: individuals are selected from the population at random with probability proportional to the value of their fitness function. At the initialization step, all candidate individuals are assigned the same value of fitness.

**Parthenogenesis**: each selected individual in the current population generates a new one. The parthenogenesis operator expands the selected individual by increasing the subsequence length of a given *γ* value, selected at random in the interval [0,Max _*β*_−*β*_*i*_+1]; *γ* is then split at random into *γ*_1_,*γ*_2_ (*γ*_1_+*γ*_2_=*γ*): the first one (*γ*_1_) is the number of positions that will be added at the head of the new subsequence, the second one (*γ*_2_) the ones to be appended at the end. The name of this operator is inspired to the biological form of reproduction in which the ovum develops into a new individual without fertilization.

**Trimming**: this operator is executed in order to reduce the length of the extracted subsequences, maintaining the separating power of the subsequence, and is applied to an individual just before it is entered in *R*. Starting from an individual *s*_1_ (*α*_1_,*β*_1_,*σ*_1_), the *trimming* operator looks for another individual *s*_2_ (*α*_2_,*β*_2_,*σ*_2_), such that *α*_1_=*α*_2_ and *σ*_1_≤*σ*_2_≤*σ*_1_+*β*_1_−*β*_2_ (i.e., *s*_2_ is a subsequence of *s*_1_, with the same discriminating power (*α*_1_=*α*_2_).

**Mutation**: Mutation is needed to prevent an irrecoverable loss of potentially useful information that parthenogenesis and trimming may occasionally cause. This operator is a random alteration of the length of an individual that occurs with small probability, and randomly cuts at the head or tail of the subsequence.

#### Steps of the algorithm

In the following, we describe the steps of the algorithm:

1. Initialize a random population *S*_*t*_,*t*=0 of individuals. Set *R*=*∅*.
2. Create a new population *S*_*t*+1_ by repeating the following steps until no new solution is found, i.e., until the solution does not change any more respect to the others:
  Through the *selection* operator, select an individual from the population;
  By means of the *parthenogenesis* genetic operator, expand the selected individual, by increasing the subsequence length of a given *γ* value and form a new individual. If no more expansion are allowed (*β*_*i*_ = Max _*β*_), the new individual is the exact copy of the old one.
  By using the *mutation* operator and according to a mutation probability, randomly cut the new solution at a random position.
  Perform the *trimming* of the new individual to reduce its *β*_*i*_.
  Check if the new individual is dominated by one in the repository *R*; if not, discard from the repository all individuals dominated by the new one and insert the new individual in *R* and in *S*_*t*+1_ and compute its fitness value.
3. Set *t*=*t*+1
4. Check termination conditions: (i) the *R* has not changed in the last *k*>10 iterations; (ii) the maximum number of iterations (Maxiter) has been reached; (iii) the number of individuals exceeds the value of Dimstore. If one of the stopping condition is satisfied, stop, and return *R*.
5. Go to the step 2.

**Fig. 1:**
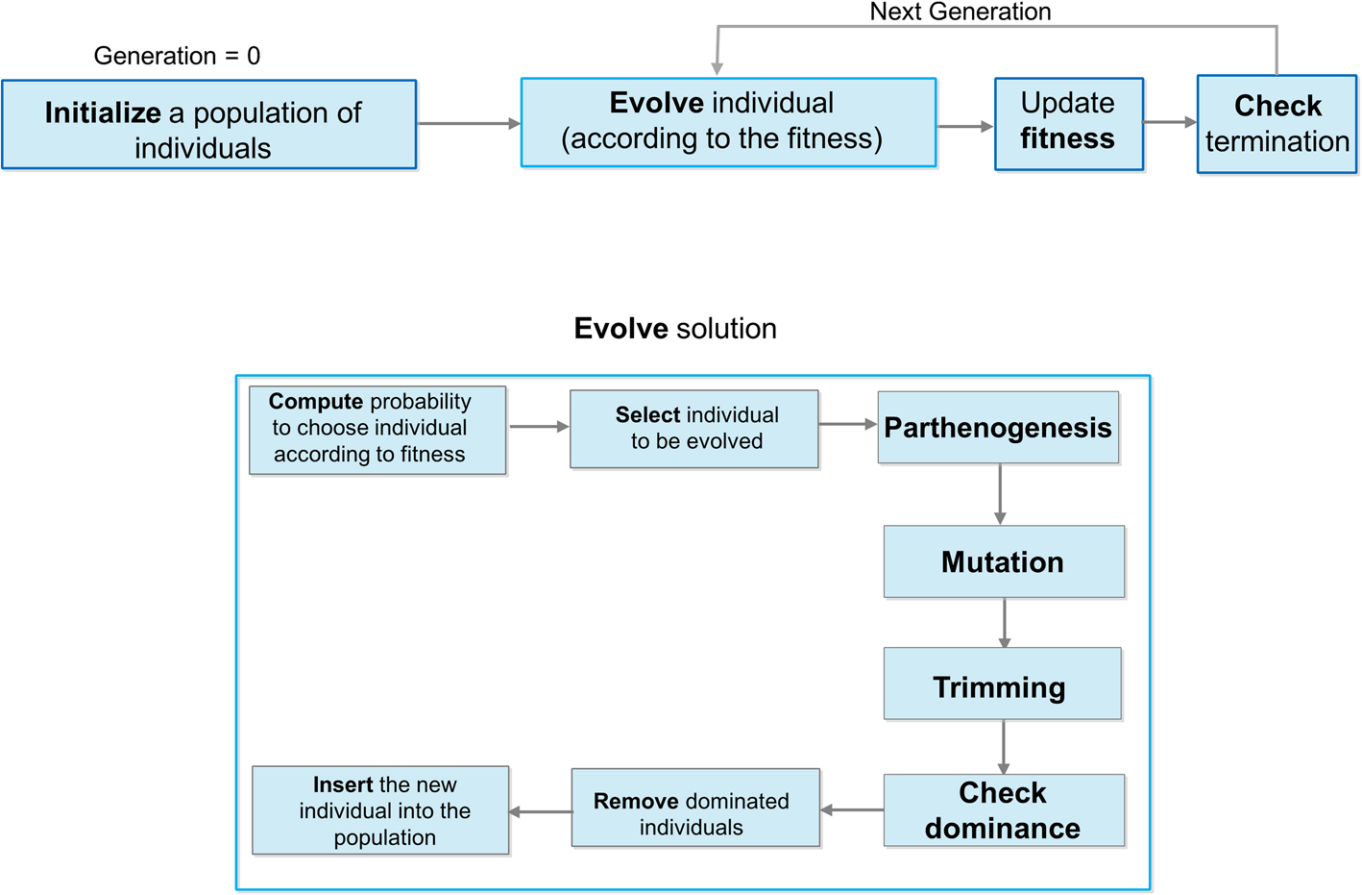
Extended flowchart of MISSEL. Graphical representation of the whole procedure implemented by the genetic algorithm: (i) initialization of a random population of individuals; (ii) evolution of the current population according to a set of *genetic operators*; (iii) evaluation of the fitness of each individual in the population according to fitness function F and updating of the fitness value; (iv) checking the termination conditions. The evolution step is further composed of probability computation, selection of individuals, parthenogenesis, mutation, trimming and dominance checking; then the individual is inserted into the current population, where a proper cleaning is also made according to dominance rules

An extended flowchart of our algorithm is drawn in Fig. 1.

#### Score computation

Once the final repository of individuals *R* is obtained, we can derive aggregate information from the individuals contained therein. By the construction of *R*, we know that *R* contains *non-dominated* and possibly *equivalent* individuals (according to definition 1, these are subsequences of the same length and the same discriminatory power that do not fully overlap over the sequence). The density of the individuals along the sequence will be used in the experiments described in the following to characterize different genomic regions of the same virus type. To bring this information to light, we compute, for each position *j* of the sequence, the ratio between the sum of the *α*_*i*_ for the individuals *s*_*i*_∈*R* that contain that position, and the similar sum over the *β*_*i*_ (see Eq. 4).

4 $$  score(j) = \frac{\sum_{s_{i} \in H_{j}}{\alpha_{i}}} {\sum_{s_{i} \in H_{j}} {\beta_{i}}}, H_{j} = \left\{s_{i} \in R: \sigma_{i} \leq j \leq \sigma_{i}+\beta_{i}\right\}, j = 1,..,n  $$

Such score measures can be charted over the length of the complete sequence to see if the discriminating power is concentrated in some portion of the sequence. When the scores are charted for different genomic regions, we are interested in comparing the landscape of discriminating power along the regions, to see where and how they concentrate or exhibit isolated peaks. Within each region, we can then point out sets of specific consecutive nucleotide positions that discriminate for the considered classes.

Finally, we store the *equivalent* and *non-dominated* solutions, measuring for each of them the effective discriminating power with a classification method, and summarizing the results according to characteristics of the individuals (e.g., their length and discriminating power).

### Classification of sequences through individuals in *R*

To evaluate the *equivalent*, *non-dominated* individuals, we adopt an automatic classifier, based on the well-known approach of *supervised learning* [51]: a classification model is computed from objects with known classes (training set), and then unknown objects (test set) are automatically assigned to a class by analyzing the classification model. An additional goal is also to compute a clear and compact classification model that fits the data, for example “if-then” rules that have been extracted by rule-based classifiers. The classification model aids biologists to extract relevant positions that are discriminant for new sequence samples [22].

Proper validation can be performed splitting the available labeled data at random into training and test sets, and computing correct classification percentages for both. An important aspect of automated classification is the prevention of overfitting, which can manifest itself with very high correct classification percentages in the training sets, and very poor ones on the test sets. Additional validation can be performed by random permutations of class labels; solutions extracted from data with randomly permuted labels should not exhibit separating power. Results from these validation analyses, which we provide for our experiments, give us additional confidence in the quality of our proposed method and in the fact that we are properly handling overfitting risks.

Our method is released as a software package called MISSEL and is available at http://dmb.iasi.cnr.it/missel.php. For installation and usage the reader may refer to the user guide provided as Additional file 1.

## Results and discussion

In this section, we present the main results of our method applied to three experimental data sets of virus sequences.

### Experimental data sets

We analyze data sets of sequences belonging to three virus groups: Influenza, Polyoma, and Rhino viruses.

**Influenza**. Influenza viruses (INL) are involved in the etiology of acute respiratory infections of the upper and lower respiratory tract. They are RNA viruses belonging to the Orthomyxoviridae family. We can distinguish Influenza virus types A, B, and C. Influenza virus types A and B cause annual epidemics in temperate climates, while Influenza virus C is less common. Influenza A epidemics follow the emergence of a novel virus resulting from genetic shift and a new combination of hemagglutinin (HA) and neuraminidase (NA) genes. Based on the antigenic differences in the neuraminidase and hemagglutinin glycoproteins, Influenza virus A is subdivided into several subtypes. New strains of Influenza virus B are instead the result of selective immune pressure that results in small antigenic changes of the hemagglutinin gene. We analyzed the sequences of the H1N1 and H3N2 subtypes of Influenza A (INL A); these subtypes co-dominated the last three influenza seasons in Europe with different distribution of cases between Northern and Southern Europe.

The Influenza viruses data set is composed of more than 40,000 sequences from the NA, HA, and MP genomic regions of H1N1 and H3N2 subtypes (serotypes) of Influenza type A. The average fragment length is 1291 nucleotides and the average number of sequences from each genomic region is greater than 6000. The reader may refer to Table 2 for additional details.

**Table 2:** Data set description of Influenza viruses

Class/genomic region	NA	HA	MP
H1N1	5999	6110	11994
H3N2	4716	4715	9427
Number of sequences	10715	10825	21421
Number of nucleotides	1410	1701	756

The number of sequences and their corresponding nucleotides is shown for each virus subtype (H1N1 and H3N2), which is considered as a different class, and for each genomic region

**Human Polyoma viruses**. Currently, 13 human polyomaviruses have been identified: BKPyV, JCPyV, KIPyV, WUPyV, MCPyV, HPyV6, HPyV7, TSPyV, HPyV9, HPyV10 (MW and MX isolates), STLPyV, and HPyV12. Members of the Polyomaviridae family are small non-enveloped DNA viruses with an icosaedral capsid which surrounds a circular double stranded DNA genome of about 5 Kb in length. The genome can be subdivided into three functional regions: the early region encoding for the large and small tumour antigens (LT-ag and ST-ag); the late region encoding for the structural proteins (VP1, VP2 and VP3); and the non-coding control region containing the origin of replication and transcriptional control elements.

The Polyoma viruses data set is composed of BKPyV, JCPyV, KIPyV, WUPyV, MCPyV, HPyV6, HPyV7, TSPyV, HPyV9, HPyV10 (MW and MX isolates), STLPyV, and HPyV12 sequences. In particular, for each human Polyoma virus five genomic regions (i.e., VP1, VP2, VP3, ST, LT) have been considered. Each genomic region is composed of more than 120 sequences, longer than 450 nucleotides, and belonging to the 13 different species. The reader may refer to Table 3 for additional details.

**Table 3:** Data set description of Polyoma viruses

Class/genomic region	VP1	VP2	VP3	ST	LT
BKPyV	26	25	25	13	26
HPyV6	7	7	7	7	7
HPyV7	7	7	7	7	7
HPyV9	2	2	2	2	2
HPyV10	1	1	1	1	1
HPyV12	2	2	2	2	2
JCPyV	23	20	21	15	21
KIPyV	10	8	8	14	8
MCPyV	3	2	2	28	13
MW	19	19	19	15	19
MX	1	1	1	1	1
STLPyV	6	6	6	6	6
WUPyV	14	23	14	16	14
Number of sequences	121	123	115	127	127
Number of nucleotides	1065	726	588	519	828

The number of sequences and their corresponding nucleotides is shown for each virus subtype, which is considered as a different class, and for each genomic region

**Human Rhino viruses**. Human Rhino viruses (HRV) are considered as the cause of the common cold, but their association with lower respiratory tract infections and with asthma inception has been recently acknowledged [52, 53]. They have been historically classified into 99 reference serotypes in the genus Rhino virus of the family Picornaviridae [54], but more than 160 genotypes are now reclassified into three species (HRV-A, B, and C) into the Enterovirus genus of the same family [55, 56]. The increasing use of molecular techniques led to identify species and genotype through phylogenetic analysis, as well as to detect associations with clinical syndromes. Most PCR-based tests for HRV detection target short conserved fragments of 5’ Untranslated Region (UTR) in order to detect the majority of HRVs in one run, but typing of HRVs requires sequencing of longer and more variable genomic regions. In several studies a large portion of the 5’UTR and/or the region coding for viral protein (VP4) and part of the VP2 have been amplified. However, genetic categorization of HRVs is complicated by a wide genetic diversity generating minor variants and novel strains and by recombination events that occurred in the evolutionary history, as in the case of HRV-C [57–60]. As an example, most HRV-C strains have a 5’UTR derived from recombination events with HRV-A and would segregate together with HRV-A in a phylogenetic analysis conducted only on the 5’UTR.

For the HRV data set, we choose the VP4 and part of the VP2 region that codes for structural viral protein, which are phylogenetically characterized in several previous studies [32, 61]. The data set is composed of 1316 sequences from the VP4/2 genomic region with an average length of 222 nucleotides, that belong to the three different species of Rhino virus (species A, B, and C). The reader may refer to Table 4 for additional details.

**Table 4:** Data set description of Rhino viruses

Class/genomic region	VP4/2
A	752
B	209
C	355
Number of sequences	1316
Number of nucleotides	369

The number of sequences and their corresponding nucleotides is shown for each virus subtype (A, B, C), which is considered as a different class, and for the VP4/2 genomic region

All the above-mentioned data sets are available at http://dmb.iasi.cnr.it/missel.php. The sequences were originally downloaded from GenBank (http://www.ncbi.nlm.nih.gov/genbank/) and have been aligned with Clustal W of the Bioedit software [62], and then manually edited. We remark that each virus data set includes several gene regions, and that only the sequences of the same gene region are aligned and of the same length.

### Experimental results

We test MISSEL on the viral genomic sequence data sets of Influenza, Polyoma, and Rhino viruses described above. The parameters used for running MISSEL are reported in Table 5. To assess overfitting of the classification models, we chose different percentage splits into training and test sets for the different data sets under analysis. In order to further validate our approach and ensure that our results are not unduly affected by overfitting, we performed random permutations of class memberships for each data set. Through these random permutations we test the null hypothesis under which MISSEL is able to extract meaningful subsequences regardless of the class partition imposed on the training set. Such hypothesis would be accepted only in the presence of a marked overfitting behavior of the algorithm. The details are reported in each subsection related to the different viral data sets. The parameter setting we selected are proven to be robust and effective to achieve reliable results. For what concerns the Max _*β*_ choice, we run MISSEL with an increasing value of *β* (up to 20) in order to find the right balance between the length of the solutions and the number of equivalent solutions for each data set. We remark that we are interested in extracting short subsequences and we chose *β* accordingly for each data set.

**Table 5:** Setting of parameters used for the execution of MISSEL

	Maxiter	Max _*β*_	Base _*σ*_	Dimstore
Influenza viruses	5·10^4^	10–20	5	10^6^
Polyoma viruses	5·10^4^	20	5	10^6^
Rhino viruses	5·10^4^	20	5	10^6^

### Influenza viruses

Table 6 reports the equivalent and non-dominated solutions for the Influenza viruses data set obtained by the algorithm. We fix the maximum solutions length to 10 for HA and NA genomic regions (i.e., *β*≤10) and to 20 for the largest MP genomic region (i.e., *β*≤20).

**Table 6:** Number of equivalent and non-dominated solutions for Influenza viruses H1N1 and H3N2 with *β*≤10 for HA and NA genomic regions and *β*≤20 for MP genomic region

Genomic region	Number of solutions
HA	655
MP	23
NA	486
Total number of solutions	1164

We provide the score value (formula 4) along the positions of the sequence, see Panels (a)-(c) of Fig. 4. Positions with high score values indicate locations of the sequence where a large number of discriminant subsequences intersect - higher values indicating portions more interesting and rich in separating power.

**Fig. 2:**
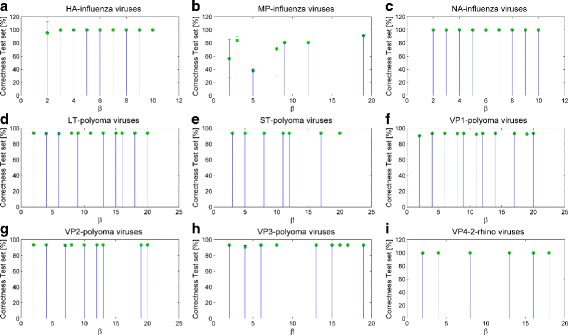
Bar plots of the classification performances on the test sets for the three analyzed types of viruses. The reported values are averaged on the solutions with the same value of *β*; the error bars refer to the corresponding standard deviations computed on all solutions. **a** HA **b** MP **c** NA data sets of Influenza viruses, **d** LT **e** ST **f** VP1 **g** VP2 **h** VP3 data sets of Polyoma viruses, and **i** VP4/2 data sets of Rhino viruses

**Fig. 3:**
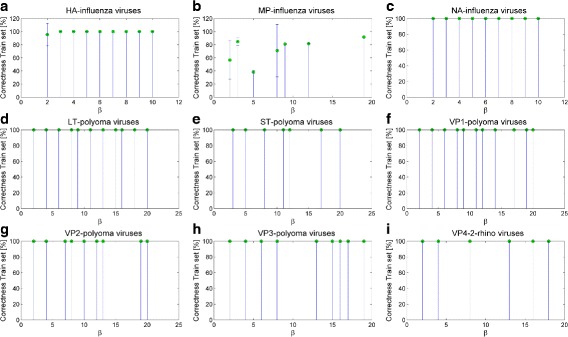
Bar plots of the classification performances on the training sets for the three analyzed types of viruses. The reported values are averaged on the solutions with the same value of *β*; the error bars refer to the corresponding standard deviations computed on all solutions. **a** HA **b** MP **c** NA data sets of Influenza viruses, **d** LT **e** ST **f** VP1 **g** VP2 **h** VP3 data sets of Polyoma viruses, and **i** VP4/2 data sets of Rhino viruses

Then, we test all the extracted classification models using a percentage split schema of 30% for training and 70% for testing for the NA and HA genomic regions, and 15% for training and 85% for testing for the MP genomic region. We choose the previously mentioned training and test percentage splits because of the high number of sequences (i.e., more than ten thousands) and in order to obtain balanced training sets of adequate size. The results are listed in Table 7, where we highlight how the classification accuracy rates stand out at an average of 99–100% for both training and test set for HA and NA genomic regions (*β*∈[1,10]). As far as the MP genomic region is concerned, we observe a higher variability of results and an average correct classification rate on training and test set of 71%. Furthermore, Panel (a)-(c) of Fig. 2 and Fig. 3 report the bar plots with the percentage of correct recognition rates, averaged on the same *β* values, and obtained on the test and training set of Influenza viruses, respectively.

**Table 7:** Classification accuracy on training and test set for the 3 genomic regions of Influenza viruses (mean ± standard deviation computed on all solutions)

Genomic region	*β*	Train [%]	Test [%]
HA	2	95.44 ± 17.14	95.46 ± 17.05
	3	99.99 ± 0.06	99.98 ± 0.08
	4	99.99 ± 0.11	99.96 ± 0.15
	5	100 ± 0.06	99.98 ± 0.09
	6	100	99.99 ± 0.04
	7	100	99.99 ± 0.04
	8	100	99.98 ± 0.05
	9	100	99.98 ± 0.06
	10	100	99.98 ± 0.07
NA	2	100 ± 0.01	99.98 ± 0.02
	3	99.98 ± 0.11	99.96 ± 0.13
	4	100	99.99 ± 0.02
	5	100	99.99 ± 0.02
	6	100	99.99 ± 0.02
	7	100	99.99 ± 0.03
	8	100	99.99 ± 0.03
	9	100	99.99 ± 0.02
	10	100	100 ± 0.01
MP	2	56.72 ± 29.09	56.41 ± 28.95
	3	84.59 ± 5.75	83.94 ± 5.48
	5	38.46 ± 2.44	38.39 ± 2.54
	8	71.15 ± 40.04	71.24 ± 40.10
	9	81.01	80.96
	12	81.51	80.72
	19	91.66	91.14

Accuracy rates of extracted equivalent and non-dominated solutions with *β*≤10

Additional validation is performed by applying the algorithm to data with random permutations of class membership. Such a test is repeated for 100 different random permutations. The average classification performances obtained from the solutions are extremely poor, never exceeding 52% in training or 38% in testing, with an empirical p-value below 0.001, thus confirming the validity of our method.

### Polyoma viruses

Table 8 lists the number of solutions that are equivalent and non-dominated. Here, we extract alternative solutions of maximum size 20 (i.e., *β*≤20) for the Polyoma viruses data set.

**Table 8:** Number of equivalent and non-dominated solutions for Polyoma viruses with *β*≤20

Genomic region	Number of solutions
LT	53
ST	17
VP1	84
VP2	22
VP3	18
Total number of solutions	194

**Fig. 4:**
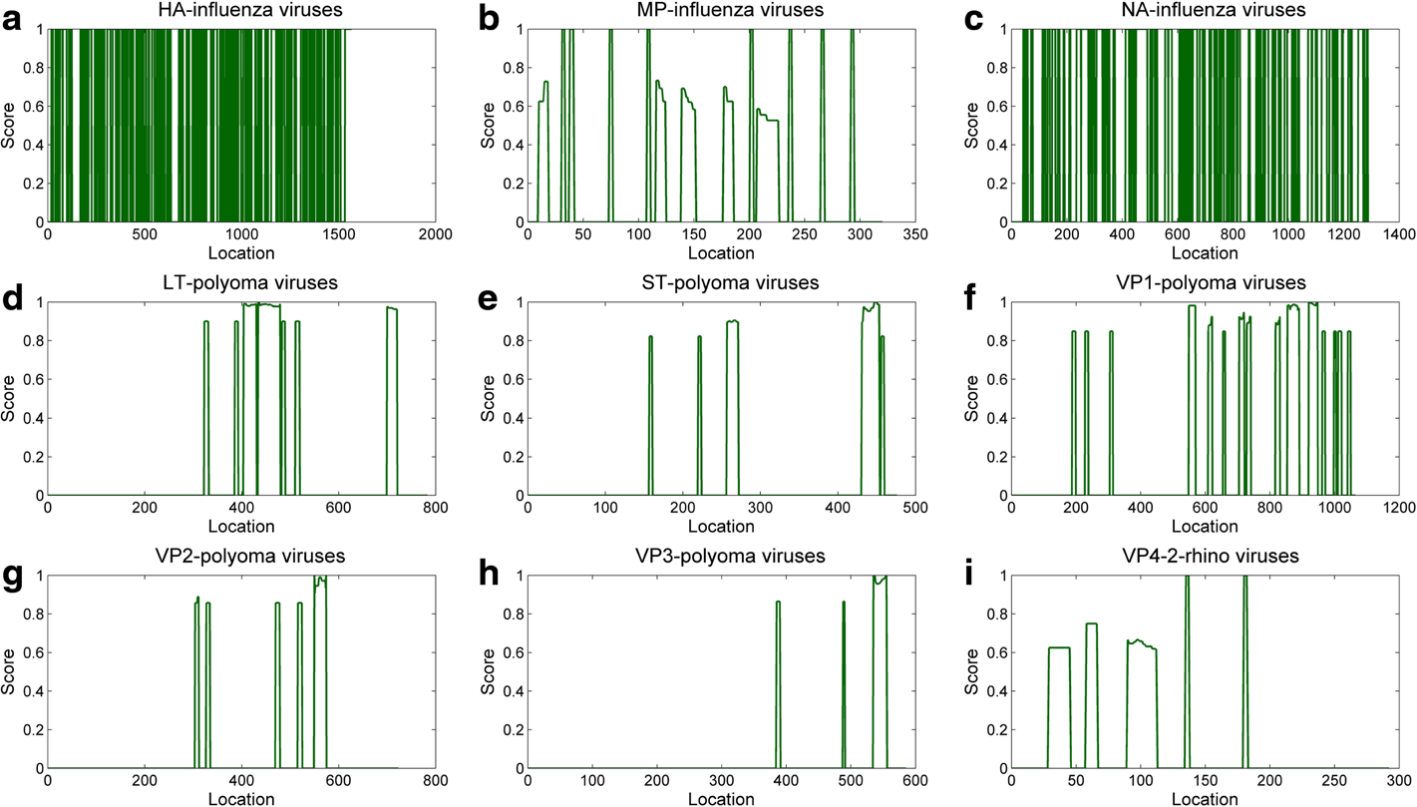
Distribution of non-dominated solutions for genomic regions: **a** HA **b** MP **c** NA of Influenza viruses, **d** LT **e** ST **f** VP1 **g** VP2 **h** VP3 of Polyoma viruses, and **i** VP4/2 of Rhino viruses

We compute the above-mentioned scores that provide a map of the solutions with respect to their location along the sequence -see panels (d)–(h) of Fig. 4.

Then, we evaluate how those solutions perform in terms of classification rate. We use a percentage split schema with 80% for training and 20% for testing. The results of the classification of alternative solutions on Polyoma viruses are reported in Table 9. Ranging *β* from 1 to 20, we obtain averaged correct classification rates of 100% on training set and 93% with very low standard deviation values on test set for all the extracted solution. The bar plots with the correct classification rate averaged on the same *β* values and obtained on the test and training set of Polyoma viruses are reported in Panels (d)-(h) of Fig. 2 and Fig. 3. When compared with the classification performance under random permutations of class memberships, the subsequences obtained on real classes result significantly different from those obtained on random ones (*p*<0.001).

**Table 9:** Classification accuracy on training and test set for the 5 genomic regions of polyomaviruses (mean ± standard deviation computed on all solutions)

Genomic region	*β*	Train [%]	Test [%]
LT	2	100	93.94
	4	100	92.93 ± 1.75
	6	100	93.18 ± 1.56
	8	100	93.94
	9	100	93.94
	11	100	93.94
	13	100	93.94
	15	100	93.94 ±1.50·10^−14^
	16	100	93.94
	18	100	93.94
	20	100	93.94 ±2.92·10^−14^
ST	3	100	93.75
	5	100	93.75
	8	100	93.75
	11	100	93.75
	12	100	93.75
	17	100	93.75
	20	100	93.75
VP1	2	100	90.32
	4	100	93.55
	6	100	93.55 ±2.97·10^−14^
	8	100	93.15 ± 1.45
	9	100	93.55
	11	100	92.38 ± 2.61
	12	100	93.55
	14	100	93.55
	17	100	93.55
	19	100	92.74 ± 2.20
	20	100	93.55
VP2	2	100	93.55
	4	100	93.55
	7	100	92.96 ± 1.31
	8	100	93.55
	10	100	93.55
	12	100	93.55
	13	100	93.55
	19	100	93.55
	20	100	93.55
VP3	2	100	93.33
	4	100	91.11 ± 1.92
	6	100	93.33
	8	100	93.33
	13	100	93.33
	15	100	93.33
	16	100	93.33
	17	100	93.33
	19	100	93.33

Accuracy rates of extracted equivalent and non-dominated solutions with *β*≤20

### Rhino viruses

Table 10 lists the number of all the equivalent and non-dominated solutions. We set the maximum solution length to 20 (i.e., *β*≤20). We compute the scores for each position of the sequence and provide a map of the sequence in Panel (i) of Fig. 4. Several peaks in score appear for Rhino virus genomic region VP4/2. We note how some areas appear much denser than others.

**Table 10:** Number of equivalent and non-dominated solutions for Rhino viruses with *β*≤20

Genomic region	Number of solutions
VP4/2 (ABC-Rhino)	11

Coming to classification performances, we use a percentage split schema of 80% for training and 20% for testing. Table 11 shows the classification results. We point out that all the alternative classification models perform with a really high reliability, i.e., an average of 100% correct classification rate on training set and 99.96% on test set, with *β*∈[1,20]. Panel (i) of Fig. 2 and Fig. 3 report the bar plots with the percentage of correct classifications averaged on the same *β* values and obtained on the test and training set of Rhino viruses, respectively.

**Table 11:** Classification accuracy on training and test set for Rhino viruses genomic region (mean ± standard deviation computed on all solutions). Accuracy rates of extracted equivalent and non-dominated solutions with *β*≤20

Genomic region	*β*	Train [%]	Test [%]
VP4/2	2	100	99.81 ± 0.27
	4	100	100
	8	100	100
	13	100	100
	16	100	100
	18	100	100

The comparison with the performance under random permutations of class memberships confirms that our method is able to identify meaningful signals in the data: correct classification rates on 100 randomly permuted instances are always below 20%, and the related p-value below 0.001.

All the results described above, as well as the extracted subsequences for each virus data set are available at http://dmb.iasi.cnr.it/missel.php and provided as Additional file 2, where the reader may also find all the specific nucleotides distinguishing the species for further investigation.

### Comparative analysis

In order to validate the results of MISSEL, we perform a comparative analysis. Since we have not found other supervised classification methods that are able to extract multiple human-readable models, we compare our method with the state-of-the art motif discovery approach, i.e., the MEME suite [63]. In particular, we select the DREME software [64], which discovers short, ungapped motifs (i.e., recurring, fixed-length patterns) that are relatively enriched in the input sequences compared with shuffled sequences or control ones. Indeed, DREME provides a list of statistically significant motifs related to each virus class. In order to fully exploit the computational approach of DREME, we set up the following experimental scheme for each virus type:

1. For each genomic region we set as input sequences the ones belonging to a given class, e.g., Rhino viruses A of VP4/2 region;
2. For each genomic region we set as control sequences the ones belonging to all the other classes, e.g., Rhino viruses B and C of VP4/2 region;
3. We set the E-value threshold (i.e., the expected number of false positives) to 10^−40^ for Influenza and Rhino viruses and to 10^−10^ for Polyoma viruses; we set this threshold very near 0 since also MISSEL is designed to discover short subsequences resulting in the accuracy range of 99% - 100%;
4. We extract the motifs that characterize each class in the considered genomic region.

The above-mentioned motifs are compared with those extracted by MISSEL that, conversely to DREME, is able to compute the species specific subsequences for all classes at once.

When considering Influenza viruses, DREME identifies a total of 290 motifs: 76 for HA, 129 for MP, and 85 for NA gene region, respectively. In this case, MISSEL extracts a larger number of subsequences (more than one thousand with an average accuracy of 99.9%) and therefore it provides the investigator with a larger number of solutions.

For Polyoma viruses, we perform the motif discovery only on gene region ST due to the large number of comparisons among the available classes that one would otherwise have to run with DREME. In this case, it identifies a total of 481 motifs. The number of the motifs extracted by MISSEL (i.e., 17) is smaller, and hence our approach allows focusing on core subsequences related to the investigated virus class. Furthermore, DREME does not find enriched short motifs for those classes with a set of under-represented sequences, conversely to MISSEL that finds solutions even for classes with a small number of sequences.

When considering Rhino viruses, DREME identifies a total of 101 motifs for VP4/2 region. The size of the motifs extracted by MISSEL is smaller (i.e., 11 subsequences), and hence our approach allows focusing on core motifs related to the investigated class of the virus.

To summarize, when dealing with a few number of classes (e.g., Influenza viruses) an approach like DREME is viable when dealing with problems with a small number of classes; when dealing with more than three classes, in order to extract a manageable number of motifs, one has to set an unrealistically high E-value threshold. Conversely, a supervised-based method like MISSEL can be preferable when addressing multiclass problems (e.g., Polyoma viruses) both from a computational point of view and in terms of classification performance.

To conclude, we wish to highlight that most of the subsequences extracted by MISSEL are different from the ones computed by state-of-the-art motif discovery methods providing additional knowledge to the investigators and enhancing the novelty of our approach. Finally, unlike motif discovery approaches, MISSEL is able to identify the discriminating subsequences, their contiguous position, and is able to locate them along the region.

## Conclusions

In this paper, we have presented a method that extracts, from a set of sequences belonging to different classes, small subsequences that contain sufficient information to discriminate among the classes. The method addresses a new problem in sequence analysis and is based on a specifically designed genetic algorithm. Data sets of sequences from viral species belonging to different types (DNA virus, positive stranded RNA virus, negative stranded fragmented RNA virus) were used to test the method.

We can draw several conclusions based on the work that has been presented, below we divide these into algorithmic conclusions, which concern MISSEL in general, and biological conclusions, which concern the virus classification applications we presented. The latter are indeed important to show the potential of the proposed method in other applications.

From the *algorithmic standpoint*, the method appears to be effective both for the quality of its solutions and for the small computational effort required. The use of a properly designed genetic algorithm seems to be the right choice for this problem, and cuts down significantly the search time. The abundance of alternative solutions for the three applications poses a challenge to experts that use supervised learning methods: how many alternative solutions of high quality can one find for a classification task? MISSEL answers this question for a specific environment and it is clearly shown by the experimental results that the identified subsequences are all of utmost quality when tested with standard classifiers. Such a fact is strongly confirmed by the tests run on randomized data, for all three cases. We highlight that the method can be applied straightforwardly to any supervised learning setting where data is described by strings on any alphabet.

From the *biological standpoint*, the results indicate several aspects worthy of further analysis and investigation that are beyond the scope of this paper, but will stimulate future research.

A first aspect is related with cardinalities of the solution sets. Influenza viruses have a much larger number of mutations associated with their different classes, while much fewer are present in Polyoma viruses and indeed very few in Rhino viruses, where the essential information needed to separate among their classes seems to be concentrated in 11 very small sequences of no more that 20 nucleotides. The differences registered for the three types of viruses in terms of number of solutions and density of the subsequences along the regions suggest a link between local density of separating subsequences and local mutation rates and evolutionary pressure. Such a link would indicate that regions with many separating subsequences are critical in virus replications and thus subject to higher selective pressure; besides, the specific locations of the regions characterized by a large relative abundance of separating sequences are indeed those where selective pressure has been stronger.

**Fig. 5:**
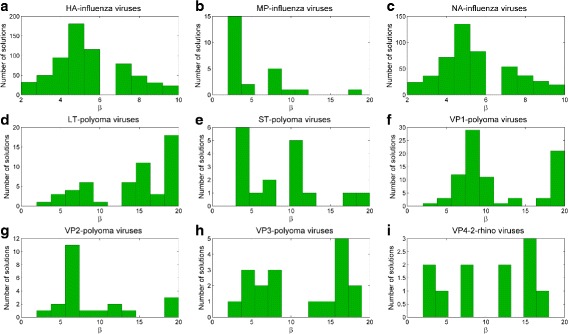
Distribution of the number of extracted solutions for each *β* value of genomic regions: **a** HA **b** MP **c** NA of Influenza viruses, **d** LT **e** ST **f** VP1 **g** VP2 **h** VP3 of Polyoma viruses, and **i** VP4/2 of Rhino viruses. The x axes report the length of solution (*β*); y axes refer to the number of extracted solutions for each *β*

**Fig. 6:**
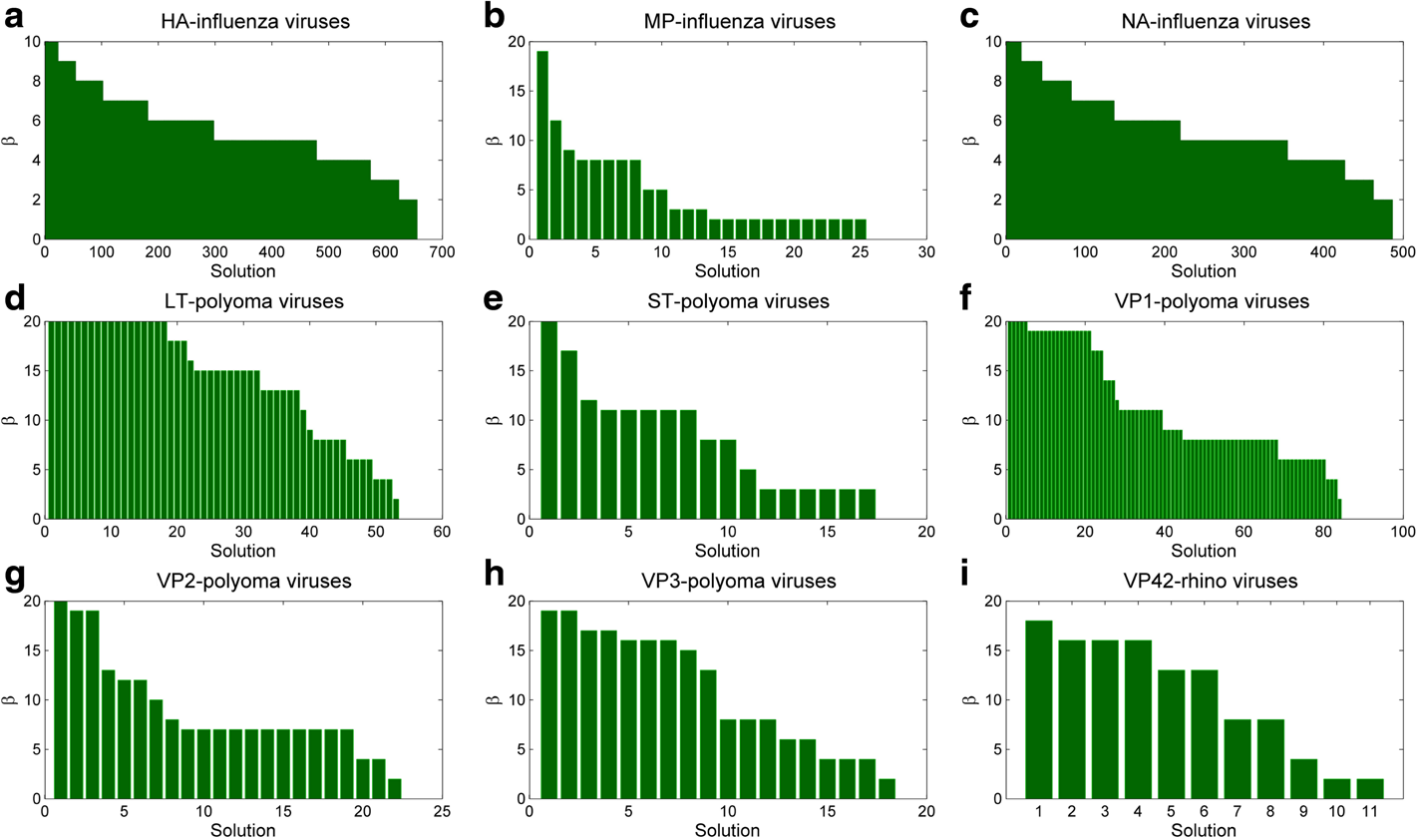
Distribution of *β* values for the whole sequences of genomic regions: **a** HA **b** MP **c** NA of Influenza viruses, **d** LT **e** ST **f** VP1 **g** VP2 **h** VP3 of Polyoma viruses, and **i** VP4/2 of Rhino viruses. The x axes report the solutions; y axes refer to the corresponding *β* value according to a decreasing order

A second interesting aspect is related to the small length (i.e., value of *β*) of the separating subsequences - summarized in Fig. 5, where we provide the number of equivalent and non-dominated subsequences identified, averaged on their length (i.e., on each *β*). An overview of the all the *β* values of each extracted solution is reported in Fig. 6, according to decreasing values of *β*. While we provide a bound to the length of the subsequences, results show that the vast majority of solutions have much smaller length.

Thirdly, length distributions vary markedly from region to region within the same virus: the skewness of the distribution exhibited by solutions in the Influenza MP region when compared with HA and NA regions, as well as the large number of solutions of maximal size for LT-Polyoma, indicate that characterizing mutations may happen in nucleotide positions, which are distant along the sequence.

Finally, the abundance of small separating subsequences may be an important support for quick and robust virus identification in settings where samples are drawn from different locations in the host body, and different - possibly unknown - virus types may be present. The availability of a well-characterized set of subsequences that can identify a virus class with respect to the other known classes would allow the construction of a reliable ensemble classifier even in the presence of unknown types.

In conclusion, we list some future extensions of the work presented in this paper. We plan to apply our method to different genomic viral sequences, as well as to more general species classification problems (e.g., DNA Barcoding [24]). It would be also interesting to extend our algorithm to deal with other classification problems within the domain of DNA, RNA or protein sequences. To conclude, it would be very useful to set up an open-access and comprehensive database comprising all separating subsequences identified in the analyzed species.

## Additional files

Additional file 1 The user guide for MISSEL software, which is a pdf file with all the instructions for the user to run the software. (PDF 891 KB)

Additional file 2 This supplementary data package includes the experimental results of MISSEL. In particular, the reader may find three folders.

1. classification_results: the spreadsheet files with the classification results for the three viral data sets (3 gene regions for Influenza, 5 gene regions for Polyoma, 1 gene region for Rhinoviruses) on training and test sets.
2. discriminating_positions: the comma separated spreadsheet files with the discriminative positions for the three viral data sets (3 gene regions for Influenza, 5 gene regions for Polyoma, 1 gene region for Rhinoviruses).
3. positions_score: the comma separated spreadsheet files with the score values on the three viral data sets (3 gene regions for Influenza, 5 gene regions for Polyoma, 1 gene region for Rhinoviruses). (RAR 292 KB)

## Abbreviations

A: Adenine
BK: B.K. (patient initials)
BKPyV: B.K. (patient initials) polyomavirus
C: Cytosine
DNA: Deoxyribonucleic acid
G: Guanine
GA: Genetic Algorithm
HRV: Human Rhino viruses
HA: Gene region Influenza hemagglutinin
HPv: Human Polyoma virus
INL: Influenza viruses
JC: John Cunningham
JCPyV: John Cunningham polyomavirus
KI: Karolinska Institute
KIPyV: Karolinska Institute polyomavirus
LT: Large t antigen
MC: Merkel cell
MCPyV: Merkel cell polyomavirus
MISSEL: Multiple SubSequences Extractor for cLassification
MP: Gene region Influenza Matrix Protein
NA: Gene region Influenza viral neuraminidase
NCCR: Noncoding control region
PML: Progressive multifocal leucoencephalopathy
ST: Small t antigen
STLPyV: Saint Louis polyomavirus
T: Thymine
TSPyV: Trichodysplasia spinulosa polyomavirus
UTR: Untranslated Region
VP1: Gene region VP1
VP2: Gene region VP2
VP3: Gene region VP3
VP4/2: Gene region viral protein VP4 and part of VP2
WU: Washington University
WUPyV: Washington University polyomavirus

## References

[1] Woese C, Fox G (1977). Phylogenetic structure of the prokaryotic domain: the primary kingdoms. PNAS.

[2] Nanney DL (1982). Dgenes and phenes in tetrahymena. Bioscience.

[3] Pace NR (1997). A molecular view of microbial diversity and the biosphere. Bioscience.

[4] Brown B, Emberson RM, Paterson AM (1999). Mitochondrial coi and ii provide useful markers for weiseana (lepidoptera, hepialidae) species identification. Bull Entomol.

[5] Bucklin A, Guarnieri M, Hill RS, Bentley AM, Kaartvedt S (1999). Taxonomic and systematic assessment of planktonic copepods using mitochondrial coi sequence variation and competitive species-specific pcr. Hydrobiology.

[6] Hebert P, Stoeckle M, Zemlak T, Francis C (2004). Identification of birds through coi dna barcodes. PLOS Biol.

[7] van Velzen R, Weitschek E, Felici G, Bakker FT (2012). Dna barcoding of recently diverged species: relative performance of matching methods. PloS one.

[8] Farris S (1972). Estimating Phylogenetic Trees from Distance Matrices James. Am Nat.

[9] Saitou N (1987). The neighbour-joining method: a new method for reconstructing phylogenetic trees. Mol Biol Evol.

[10] Munch K, Boomsma W, Huelsenbeck JP, Willerslev E, Nielsen R (2008). Statistical assignment of dna sequences using bayesian phylogenetics. Syst Biol.

[11] Altschul SF, Madden TL, Schäffer AA, Zhang J, Zhang Z, Miller W, Lipman DJ (1997). Gapped blast and psi-blast: a new generation of protein database search programs. Nucleic Acids Res.

[12] Austerlitz F, David O, Schaeffer B, Bleakley K, Olteanu M, Leblois R, Veuille M, Laredo C (2009). Dna barcode analysis: a comparison of phylogenetic and statistical classification methods. BMC Bioinforma.

[13] Meier R, Shiyang K, Vaidya G, Ng PK (2006). Dna barcoding and taxonomy in diptera: a tale of high intraspecific variability and low identification success. Syst Biol.

[14] DasGupta B, Konwar KM, Măndoiu I, Shvartsman AA (2005). Dna-bar: distinguisher selection for dna barcoding. Bioinformatics.

[15] Weitschek E, Velzen R, Felici G, Bertolazzi P (2013). Blog 2.0: a software system for character-based species classification with dna barcode sequences. what it does, how to use it. Mol Ecol Res.

[16] Sarkar IN, Planet PJ, Desalle R (2008). caos software for use in character-based dna barcoding. Mol Ecol Res.

[17] Little DP (2011). Dna barcode sequence identification incorporating taxonomic hierarchy and within taxon variability. PLoS ONE.

[18] Liu C, Liang D, Gao T, Pang X, Song J, Yao H, Han J, Liu Z, Guan X, Jiang K (2011). Ptigs-idit, a system for species identification by dna sequences of the psba-trnh intergenic spacer region. BMC Bioinforma.

[19] Albu M, Nikbakht H, Hajibabaei M, Hickey DA (2011). The dna barcode linker. Mol Ecol Res.

[20] Kuksa P, Pavlovic V (2009). Efficient alignment-free dna barcode analytics. BMC Bioinforma.

[21] Weitschek E, Cunial F, Felici G (2015). Laf: Logic alignment free and its application to bacterial genomes classification. BioData Mining.

[22] Tan P, Steinbach M, Kumar V (2005). Introduction to Data Mining.

[23] Kotsiantis SB, Zaharakis I, Pintelas P. Supervised machine learning: A review of classification techniques. In: Proceedings of the 2007 Conference on Emerging Artificial Intelligence Applications in Computer Engineering: 2007. p. 3–24.

[24] Weitschek E, Fiscon G, Felici G (2014). Supervised DNA Barcodes species classification: analysis, comparisons and results. BMC BioData Min.

[25] Lavigne R, Darius P, Summer EJ, Seto D, Mahadevan P, Nilsson AS, Ackermann HW, Kropinski AM (2009). Classification of myoviridae bacteriophages using protein sequence similarity. BMC Microbiol.

[26] Bao Y, Chetvernin V, Tatusova T (2012). Pairwise sequence comparison (pasc) and its application in the classification of filoviruses. Viruses.

[27] Weitschek E, Presti AL, Drovandi G, Felici G, Ciccozzi M, Ciotti M, Bertolazzi P (2012). Human polyomaviruses identification by logic mining techniques. Virol J.

[28] Muhire B, Martin DP, Brown JK, Navas-Castillo J, Moriones E, Zerbini FM, Rivera-Bustamante R, Malathi V, Briddon RW, Varsani A (2013). A genome-wide pairwise-identity-based proposal for the classification of viruses in the genus mastrevirus (family geminiviridae). Arch Virol.

[29] Hara K, Rivera MM, Koh C, DeMino M, Page S, Nagabhyru PR, Rehermann B, Liang TJ, Hoofnagle JH, Heller T (2014). Sequence analysis of hepatitis c virus from patients with relapse after a sustained virological response: relapse or reinfection?. J Infect Dis.

[30] Kaji M, Watanabe A, Aizawa H (2003). Differences in clinical features between influenza a h1n1, a h3n2, and b in adult patients. Respirology.

[31] Midulla F, Pierangeli A, Cangiano G, Bonci E, Salvadei S, Scagnolari C, Moretti C, Antonelli G, Ferro V, Papoff P (2012). Rhinovirus bronchiolitis and recurrent wheezing: 1-year follow-up. Eur Respir J.

[32] Pierangeli A, Ciccozzi M, Chiavelli S, Concato C, Giovanetti M, Cella E, Spano L, Scagnolari C, Moretti C, Papoff P (2013). Molecular epidemiology and genetic diversity of human rhinovirus affecting hospitalized children in rome. Med Microbiol Immunol.

[33] Colorni A, Dorigo M, Maniezzo V (1992). An investigation of some properties of anant algorithm. Proceedings Of The Parallel Problem Solving From Nature Conference (Ppsn 92).

[34] Holland JH (1975). Adaptation in Natural and Artificial Systems: An Introductory Analysis with Applications to Biology, Control, and Artificial Intelligence.

[35] Kirkpatrick S, Gelatt CD, Vecchi MP (1983). Optimization by simmulated annealing. Science.

[36] Glover F (1989). Tabu search-part i. ORSA J Comput.

[37] James K, Russell E (1995). Particle swarm optimization. Proceedings of 1995 IEEE International Conference on Neural Networks.

[38] Li X (2003). A non-dominated sorting particle swarm optimizer for multiobjective optimization. Genetic and Evolutionary Computation—GECCO 2003.

[39] Handl J, Kell DB, Knowles J (2007). Multiobjective optimization in bioinformatics and computational biology. IEEE/ACM Trans Comput Biol Bioinforma (TCBB).

[40] Michailides C, Angelides DC (2015). Optimization of a flexible floating structure for wave energy production and protection effectiveness. Eng Struct.

[41] Deb K, Reddy AR (2003). Reliable classification of two-class cancer data using evolutionary algorithms. BioSystems.

[42] Miettinen K (1999). Nonlinear Multiobjective Optimization vol. 12.

[43] Browne WJ, Goldstein H, Rasbash J (2001). Multiple membership multiple classification (mmmc) models. Stat Model.

[44] Maris E (1999). Estimating multiple classification latent class models. Psychometrika.

[45] Liu J, Iba H. Selecting informative genes using a multiobjective evolutionary algorithm. In: Evolutionary Computation, 2002. IEEE: 2002. p. 297–302.

[46] Kohavi R, John GH (1997). Wrappers for feature subset selection. Artif Intell.

[47] Bertolazzi P, Felici G, Festa P, Fiscon G, Weitschek E (2016). Integer programming models for feature selection: New extensions and a randomized solution algorithm. Eur J Oper Res.

[48] Festa P, Resende MGC (2009). Hybrid GRASP heuristics. Stud Comput Intell.

[49] Festa P, Resende MGC (2011). GRASP: Basic components and enhancements. Telecommun Syst.

[50] Bertolazzi P, Felici G, Festa P, Lancia G (2008). Logic classification and feature selection for biomedical data. Comput Math Appl.

[51] Dulli S, Furini S, Peron E (2009). Data Mining.

[52] Kaiser L, Aubert JD, Pache JC, Deffernez C, Rochat T, Garbino J, Wunderli W, Meylan P, Yerly S, Perrin L (2006). Chronic rhinoviral infection in lung transplant recipients. Am J Respir Crit Care Med.

[53] Jackson JL, Lesho E, Peterson C (2000). Zinc and the common cold: a meta-analysis revisited. J Nutrition.

[54] Hamparian V, Colonno R, Cooney M, Dick E, Gwaltney Jr J, Hughes J, Jordan Jr W, Kapikian A, Mogabgab W, Monto A (1987). A collaborative report: rhinoviruses–extension of the numbering system from 89 to 100. Virology.

[55] Tapparel C, Junier T, Gerlach D, Cordey S, Van Belle S, Perrin L, Zdobnov EM, Kaiser L (2007). New complete genome sequences of human rhinoviruses shed light on their phylogeny and genomic features. BMC Genomics.

[56] Carstens E (2010). Ratification vote on taxonomic proposals to the international committee on taxonomy of viruses (2009). Arch Virol.

[57] Huang T, Wang W, Bessaud M, Ren P, Sheng J, Yan H, Zhang J, Lin X, Wang Y, Delpeyroux F (2009). Evidence of recombination and genetic diversity in human rhinoviruses in children with acute respiratory infection. PLoS One.

[58] Wisdom A, Leitch EM, Gaunt E, Harvala H, Simmonds P (2009). Screening respiratory samples for detection of human rhinoviruses (hrvs) and enteroviruses: comprehensive vp4-vp2 typing reveals high incidence and genetic diversity of hrv species c. J Clin Microbiol.

[59] McIntyre CL, Leitch ECM, Savolainen-Kopra C, Hovi T, Simmonds P (2010). Analysis of genetic diversity and sites of recombination in human rhinovirus species c. J Virol.

[60] McIntyre CL, Knowles NJ, Simmonds P (2013). Proposals for the classification of human rhinovirus species a, b and c into genotypically assigned types. J Gen Virol.

[61] Broberg E, Niemela J, Lahti E, Hyypia T, Ruuskanen O, Waris M (2011). Human rhinovirus associated severe pneumonia in a neonate. J Clin Virol.

[62] Hall TA (1999). Bioedit: a user-friendly biological sequence alignment editor and analysis program for windows 95/98/nt. Nucleic Acids Symposium Series, vol. 42.

[63] Bailey TL, Boden M, Buske FA, Frith M, Grant CE, Clementi L, Noble WS (2009). MEME SUITE: tools for motif discovery and searching. Nucleic Acids Res.

[64] Bailey TL (2011). Dreme: motif discovery in transcription factor chip-seq data. Bioinformatics.

