# Supplementary material for: MISSEL: a method to identify a large number of small species-specific genomic subsequences and its application to viruses classification
Source: BioData Min. 2016 Dec 6;9:38. doi: 10.1186/s13040-016-0116-2 (PMC5139023; doi:10.1186/s13040-016-0116-2)
Supplement: Additional file 1 — The user guide for MISSEL software, which is a pdf file with all the instructions for the user to run the software. (PDF 891 KB) [file 13040_2016_116_MOESM1_ESM.pdf]

MISSEL

User guide

Giulia Fiscon, Emanuel Weitschek, Paola Bertolazzi, Giovanni Felici

Contents

Introduction 2
Installation 2
Linux 2
Java 2
MISSEL package 2
MISSEL source code 2
Usage 3
Input 3
Executing MISSEL 3
Output 3
Web site 4
Citations 4
Contacts 4

Introduction

This user guide is intended for all the users that want to learn how to use MISSEL for extracting equivalent classification solutions (i.e., subsequences) from a set of genomic sequences. Please refer also to the `missel_readme.txt` file (that is included in the software package) for additional details.

Installation

Linux

MISSEL works in a Linux environment. In particular it is tested on Ubuntu 64 bit 14.04 LTS (Debian based Linux).

Java

MISSEL requires a working JAVA Virtual Machine (VM) installed. Thus, if not done yet, first download and install the free Oracle JAVA Runtime Environment from http://www.java.com/getjava/.

Several versions for the most common operating systems are available (e.g., Linux, MacOsX). Please choose the right version according to your operating system.

MISSEL package

You can download and unzip the MISSEL software package for Ubuntu (or Debian based) Linux 64 bit from http://dmb.iasi.cnr.it/missel.php (MISSEL_software_package_linux_64.zip).

MISSEL source code

Additionally, the source code is released and available at http://dmb.iasi.cnr.it/missel.php (MISSEL_source_code.zip). This can be compiled on any Linux-based operating system. For compiling the MISSEL package download the source code available at http://dmb.iasi.cnr.it/missel.php (MISSEL_source_code.zip).

Unzip the archive, enter directory “Src” and run the command "make" under Linux or Mac/OS (or any other unix-based operating system).

Additionally, MISSEL requires the program lsqcc from the Leibniz System available at http://www.utdallas.edu/~klaus/Leibnizprogram/leibnizmain.html. Please download it (http://www.utdallas.edu/~klaus/Leibnizprogram/leibniz.complete.zip) and follow instruction for its compilation and installation (http://www.utdallas.edu/~klaus/Leibnizprogram/installation.pdf).

Usage

Input

The input of MISSEL is a standard FASTA format file (.fas) of the DNA/RNA sequences to be analyzed. The heading line of each sequence is composed by the starting character ">" and the "specimen id" or "species name field" separated by the pipe character "|" (eg: ">polyomaLT01 | BK"). The fasta files of the analyzed data sets are available at http://dmb.iasi.cnr.it/missel.php in section “Data sets”.

Executing MISSEL

Go to the directory where you extracted or compiled the MISSEL software package and execute following command from a prompt: `./runMissel Input.fas`
e.g., ./runMissel VP42.fas

Output

You will find the output files in N output Directories, where N represents the number of equivalent non dominated solutions extracted by the algorithm.
In each directory you can find:

- classification rates for training and test sets (.stats.csv)
- confusion matrices for training and test sets (.confmatrix.csv)
- classification formulas (.formulas.csv)
- extracted features (.features)
- solution info (.sol)
- additional scores and information that characterize the solution (.csv)

Additionally, the program prints the number of equivalent non dominated solutions and creates in the execution directory a comma separated spreadsheet file (called *Input*.stats.csv) with the summary of the classification results of all the extracted solutions, e.g., VP42.stats.csv, and a comma separated spreadsheet file (called *Input*_positions.csv) with the discriminative positions for the input data sets (see supplementary data for examples of these output files).

Advanced Parameters

For advanced program settings, please edit the file parameters.dat

- BETA - the length of the solution
- GRASPITER - the maximum number of iterations (maxiter)
- PERCSLICING - the percentage split ratio for training set

Web site

The web site http://dmb.iasi.cnr.it/missel.php contains an up-to-date releases and experimental data and results of MISSEL.

Citations

If you use MISSEL please cite:

Giulia Fiscon, Emanuel Weitschek, Eleonora Cella, Alessandra Lo Presti, Marta Giovanetti, Muhammed Babakir-Mina, Marco Ciotti, Alessandra Pierangeli, Massimo Ciccozzi, Paola Bertolazzi and Giovanni Felici: **MISSEL: A method to identify characterizing small genomic subsequences and its application for viruses classification.**

Contacts

Please contact Giulia Fiscon (giulia.fiscon@iasi.cnr.it) or Emanuel Weitschek (emanuel@iasi.cnr.it) for comments and questions.
